# Molecular Characterization and Clinical Relevance of Lysine Acetylation Regulators in Urological Cancers

**DOI:** 10.3389/fonc.2021.647221

**Published:** 2021-05-31

**Authors:** Jian Zhang, Chunning Zhang, Huali Jiang, Hualong Jiang, Yawei Yuan

**Affiliations:** ^1^ Department of Radiation Oncology, Affiliated Cancer Hospital & Institute of Guangzhou Medical University, State Key Laboratory of Respiratory Diseases, Guangzhou Institute of Respiratory Disease, Guangzhou, China; ^2^ The First Tumor Department, Maoming People’s Hospital, Maoming, China; ^3^ Department of Cardiovascularology, Tungwah Hospital of Sun Yat-sen University, Dongguan, China; ^4^ Department of Urology, Tungwah Hospital of Sun Yat-sen University, Dongguan, China

**Keywords:** lysine acetyltransferase, lysine deacetylase, urological cancers, genetic alterations, cancer pathways

## Abstract

**Background:**

Lysine acetylation and deacetylation are posttranslational modifications that are able to link extracellular signals to intracellular responses. However, knowledge regarding the status of lysine regulators in urological cancers is still unknown.

**Methods:**

We first systematically analyzed the genetic and expression alterations of 31 lysine acetylation regulators in urological cancers. The correlation between lysine acetylation regulators and activation of cancer pathways was explored. The clinical relevance of lysine acetylation regulators was further analyzed.

**Results:**

We identified that there are widespread genetic alterations of lysine acetylation regulators, and that their expression levels are significantly associated with the activity of cancer hallmark-related pathways. Moreover, lysine acetylation regulators were found to be potentially useful for prognostic stratification. HDAC11 may act as a potential oncogene in cell cycle and oxidative phosphorylation of urological cancers.

**Conclusion:**

Lysine acetylation regulators are involved in tumorigenesis and progression. Our results provide a valuable resource that will guide both mechanistic and therapeutic analyses of the role of lysine acetylation regulators in urological cancers.

## Introduction

Acetylation is the most common type of post-translational modification (PTM) of proteins, and it plays crucial roles in the development and progression cancer (1–3). Lysine acetylation is a reversible epigenetic PTM that plays crucial roles in the eukaryotic cells, which is regulated by the antagonistic actions of two families of enzymes: lysine acetyltransferases (KATs) and lysine deacetylases (KDACs) (4–6). Protein lysine acetylation and deacetylation contribute to several processes that maintain the proper functioning of cells, including transcriptional regulation and metabolic functions. Therefore, acetylation and deacetylation by lysine acetylation regulators has emerged as a crucial PTM for a wide range of cellular processes and is involved in aging and the development of several diseases, including cancer (7, 8). In addition, acetylation of lysine residues mediated by these regulators has been shown to be involved in the development of several diseases (6, 9, 10). Thus, a comprehensive understanding of the genetic alterations and expression perturbations underlying cancer cell heterogeneity is necessary to elucidate protein acetylation-based therapeutic targets.

Urological cancers entail the management of prostate, bladder, kidney, and testis cancer. The Global Burden of Disease Study showed a 2.1-fold increase in kidney cancer, a 1.5-fold increase in bladder cancer, and a 3.2-fold increase in prostate cancer  (11). Women comprise 23.2% of new cases and 27.4% of deaths for bladder cancer and 34.7% of new cases and 33.1% of deaths for kidney cancer (12). Aberrant acetylation and deacetylation of genes were involved in occurrence and development of tumor, especially urological cancers (13–15). However, the molecular alterations and clinical prognostic value of lysine acetylation regulators in urological cancers are still unclear.

In this study, we aimed to systematically characterize the molecular alterations and clinical relevance of lysine acetylation regulators in urological cancers. We identified that there exist widespread genetic alterations (including genetic mutations and copy number variations) in lysine acetylation regulators among urological tumors. We also assessed whether perturbations in the expression of lysine acetylation regulators was correlated with the activity of cancer pathways. Moreover, we further explored the clinical prognostic value of lysine acetylation regulators, and found that lysine acetylation regulators are potentially useful markers for prognostic stratification. Our analysis indeed the importance of lysine acetylation regulators in urological cancers development, and lays a foundation for the development of therapeutic strategies based on lysine acetylation.

## Methods

### Collection of Lysine Acetylation Regulators

**Figure 1:**
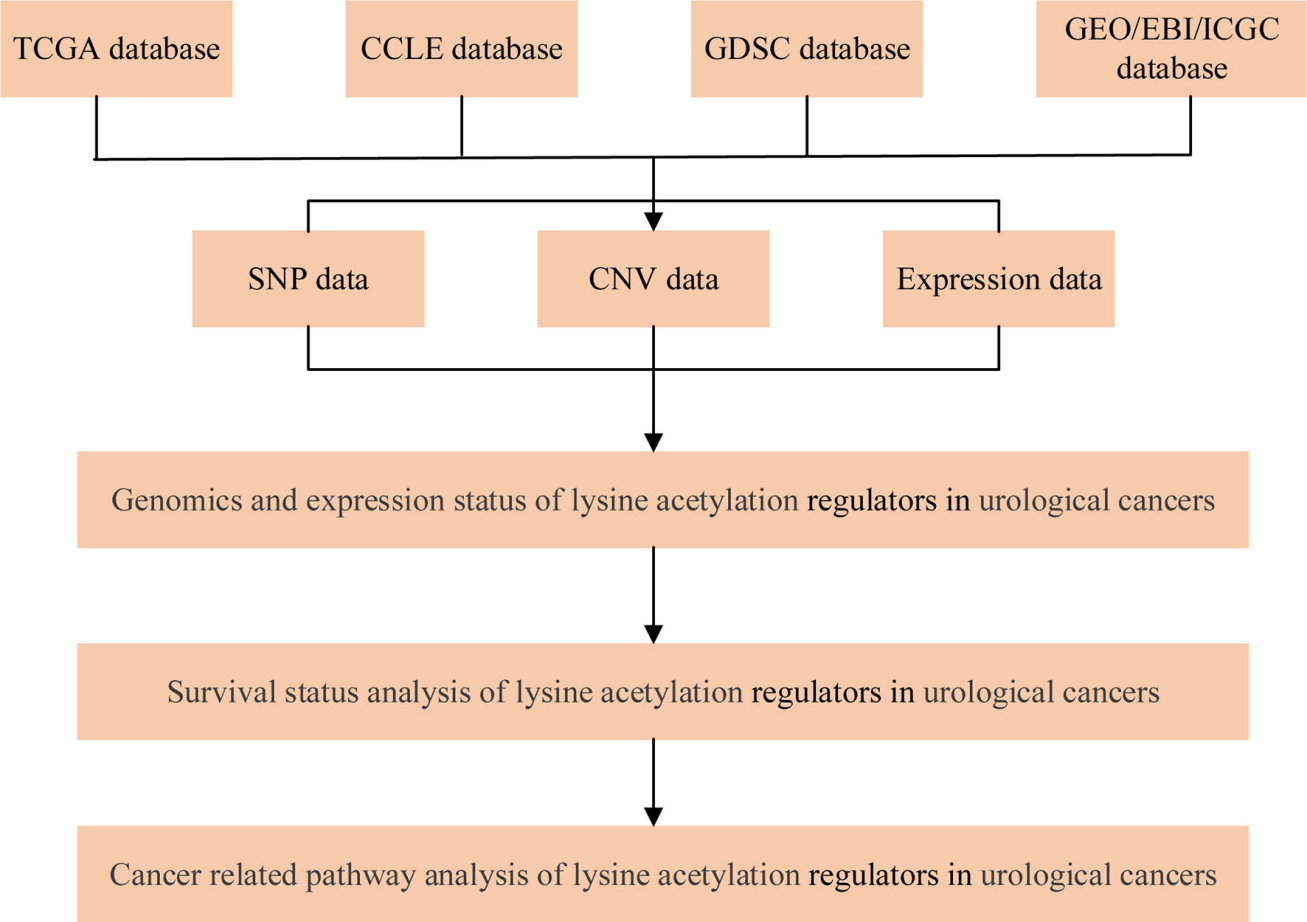
A schematic diagram for study design.

A flowchart of the study design is shown in **Figure 1**. 31 lysine acetylation regulators were collected from recently published review papers (16, 17), including 13 KATs and 18 KDACs. All these gene symbols were converted into Ensemble gene IDs and HGNC symbols by manually curated from GeneCards (https://www.genecards.org/).

### Genome-Wide Omics Data Across Six Cancer Types

The omics datasets were downloaded from TCGA (http://cancergenome.nih.gov/). TCGA projects of six urological cancers, including kidney renal clear cell carcinoma (KIRC), kidney renal papillary cell carcinoma (KIRP), kidney chromophobe (KICH), bladder urothelial carcinoma (BLCA), prostate adenocarcinoma (PRAD) and testicular germ cell tumors (TGCT). All the somatic mutation data were obtained from TCGA database. The copy number variation data were downloaded from Broad GDAC Firehose (https://gdac.broadinstitute.org/). GISTIC was used to identify genomic regions that are significantly gained or lost across a set of tumors (18). RNA-seq data were obtained from the TCGA project *via* the R-package “TCGAbiolinks” (19), which is specifically developed for integrative analysis with GDC data. The clinical information for patients of urological cancer types were downloaded from TCGA project *via* the R-package “TCGAbiolinks”.

### Genomic, Transcriptomic Data of Lysine Acetylation Regulators Across Cell Lines and Cancers

Genome-wide mutation data across cell lines were collected from the Broad Institute Cancer Cell Line Encyclopedia (CCLE) and the Genomics of Drug Sensitivity in Cancer database (GDSC) (20, 21). The cell lines were classified into different cancer types based on their annotations. In total, there were 14 cell lines across 2 cancer types from CCLE and 26 cell lines across 3 cancer types from GDSC. The mutation frequency of lysine acetylation regulators in each cancer type was defined as the proportion of cell lines with the regulator mutations. In addition, we also downloaded the copy number variation data for cell lines from CCLE and GDSC. There were 37 cell lines across 2 cancer types in CCLE and 47 cell lines in GDSC with CNV data. We calculated the CNV frequency in each cancer types as the proportion of cell lines with CNV amplification and deletion.

To validate the expression of lysine acetylation regulators across cancer types, we collected gene expression data across 778 samples representing 4 cancer types. These data were collected from Gene Expression Omnibus (GEO). To minimize inter-platform variation, only datasets generated from the Affymetrix Human Genome U133 Plus 2.0 Array were processed to develop the meta-dataset. Each dataset was preprocessed with RMA normalization, merged, and batch effect-corrected *via* Combat method (22).

### Identification of Differentially Expressed Genes

To identify differentially expressed genes in each cancer type, we used the Wilcox’s rank sum test to identify differentially expressed genes. Genes with at least two-fold changes or less than half-fold changes and adjusted p-values <0.05 in expression were identified as differentially expressed genes using R package limma.

#### Immunohistochemistry Analysis

To validate the protein expression of differentially expressed genes and activity of cell cycle and oxidative phosphorylation related pathway, as per the method described by our previous study (23), the protein of EP300, cyclin dependent kinases 2 (CDK2), cyclin A2 (CCNA2), NADH ubiquinone oxidoreductase complex assembly factor 8 of complex I (NDUFB8) and succinate dehydrogenase complex iron sulfur subunit B of complex II (SDHB) in TGCT, kidney_Tumor, BLCA and PRAD were clarified by immunohistochemistry analysis. All captured images were manually annotated by certified pathologists.

### Oncogenic Pathway Activity Across Cancer Types

To calculate the activity of cancer hallmark-related pathways, the FPKM-based gene expression was first transformed to Z-score by zFPKM package. To further estimate variation of gene set enrichment through the samples of an expression data set, the normalized gene expression were administered to Gene Set Variation Analysis (GSVA) (24). To identify the lysine acetylation regulators that were correlated with activation or inhibition of pathway, we calculated the Pearson Correlation Coefficient (PCC) between expression of lysine acetylation regulators and pathway activity. The regulator-pathway pairs with |PCC|>0.5 and adjusted p-value<0.01 were identified as significantly correlated lysine acetylation regulators.

### Clinical Relevance of Lysine Acetylation Regulators

To explore whether the expression of lysine acetylation regulators was associated with patient survival, we divided all the patients into two groups based on the median expression of HDAC9. The log-rank test was used to test the difference survival rates between two groups. This process was performed by the survival package in R program (https://cran.r-project.org/web/packages/survival/index.html). The p-values <0.05 were considered as significant.

### Validating the Clinical Association of Lysine Acetylation Regulators

We validated the clinical association of HDAC11 based on KIRC datasets from TCGA project. Patients were also divided into two groups based on the median expression of HDAC11, and the survival difference was tested by log-rank test.

### Validation of Lysine Acetylation Regulator-Pathway Correlation

To validate the lysine acetylation regulator-pathway correlation, we manually curated the TCGA database and collected KIRC gene expression data. The GSEA software tool (http://software.broadinstitute.org/gsea/index.jsp) was used to identify KEGG pathways (MSigDB, version 4.0) that show an overrepresentation of up- or downregulated genes between HDAC11 high expression and low expression. Briefly, an enrichment score was calculated for hallmark gene sets by ranking each gene and recording the maximum deviation from zero as the enrichment score.

### Statistical Analysis

Statistical analyses were performed using SPSS 17.0 (SPSS Inc., Chicago, IL, USA). All data shown are representative of at least three independent experiments, and values are expressed as the mean ± SD. Differences between two groups were analyzed using the two-tailed unpaired Student’s *t*-test; *P*< 0.05 was considered significant.

## Results

### Widespread Genetic Alterations of Lysine Acetylation Regulators Across Cancer Types

**Figure 2:**
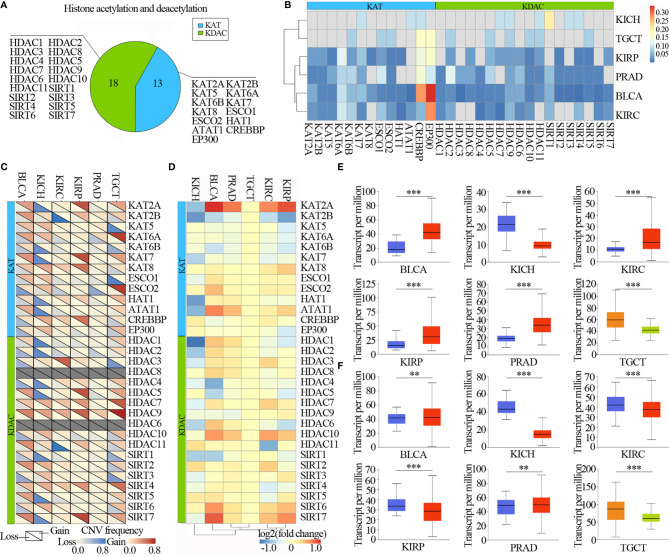
Urilogical tumors genetic and expression alterations of 31 lysine acetylation regulators. **(A)** The proportion of lysine acetylation regulators. **(B)** The mutation frequency of lysine acetylation regulators in 6 cancer types. **(C)** The CNV alteration frequency of lysine acetylation regulators in 6 cancer types. **(D)** The gene expression alterations of lysine acetylation regulators in TCGA database. **(E, F)** Box plots showing the expression distribution of KAT2A **(E)** and HAT1 **(F)** across tumor and normal samples in 6 cancer types. The blue color represents normal sample, the red color represents tumor sample, the blue color represents seminoma and the light green represents non-seminoma. **P < 0.01; ***P < 0.001.

The numbers of lysine acetylation regulators have been identified from functions and mechanisms of non-histone protein acetylation, and they can be broadly classified two groups: KATs and KDACs. We reviewed the literature and curated a catalog of 31 genes that function mainly as regulators of lysine acetylation, including 13 KATs and 18 KDACs (**Figure 2A**). We first determined the prevalence of lysine acetylation regulator alterations across 6 urological cancer types by integrating data on somatic mutations and copy number variations (CNVs). The overall average mutation frequency of lysine acetylation regulators was low, ranging from 0.0055-0.3443 (**Figure 2B** and **Table S1**). Cancer types with a higher global mutation burden (such as BLCA and KIRC) also exhibited a higher mutation frequency in lysine acetylation regulators. We identified that EP300 and CREBBP showed higher mutation frequencies (**Figure 2B**). Moreover, we found that lysine acetylation regulators in TGCT and KICH exhibited relatively few mutations compared to other cancers. Next the mutation data for 14 cell lines across 2 cancers from the Cancer Cell Line Encyclopedia (CCLE) and 26 cell lines across 3 cancers from the Genomics of Drug Sensitivity in Cancer (GDSC) database were collected. We identified that CREBBP had relatively high mutation frequencies across cancer types (**Figure S1** and **Tables S2**, **3**).

The CNV alteration frequency for all lysine acetylation regulators, and found that CNV alterations are prevalent in urological cancers. CNV analysis showed that the CNV number of KAT2A, KAT2B, KAT7, KAT8, HDAC5, HDAC7, HDAC9, SIRT4 and SIRT7 were significantly increased in TCGT, KIRP, PRAD, KIRC and BLCA, while decreased in KICH. HDAC9 and SIRT7 showed widespread CNV amplification across cancer types (**Figure 2C**). While, ESCO2 had prevalent CNV deletions. Similarly, there were also prevalent CNV alterations in lysine acetylation regulators across cell lines (**Figure S2**). To further know whether these genetic alterations affect the expression of lysine acetylation regulators, we therefore analyzed the expression of lysine acetylation regulators across 6 cancer types. We found that CNV alterations are most likely one of the prominent mechanisms leading to perturbations in the expression of lysine acetylation regulators (**Figure 2D**).

**Figure 3:**
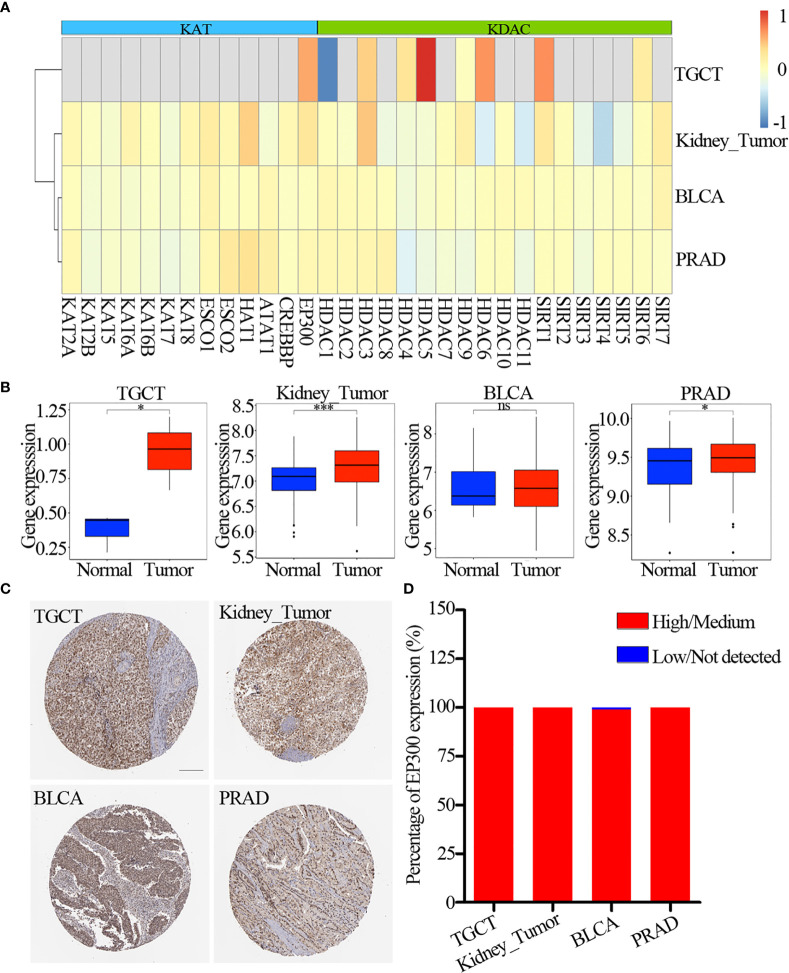
Expression of lysine acetylation regulators in GEO and EBI database. **(A)** Fold change of dysregulation genes. Red color represents upregulation genes and blue color represents downregulation genes. **(B)** The expression of EP300 in four cancer types. The blue color represents normal sample, the red color represents tumor sample. **(C)** Immunohistochemistry images of EP300 in TGCT, kidney_tumor, BLCA and PRAD. Scar bar = 200um. **(D)** Protein expression percentage of EP300 analyzed by immunohistochemistry. ns means not significant. *P < 0.05; ***P < 0.001.

The lysine acetylation regulators with CNV amplification showed significantly higher expression in cancer cells when compared to normal cells (e.g. KAT2A and ATAT1), while the regulators with CNV deletion showed significantly lower expression (e.g. SIRT6 and SIRT7). Meanwhile, we identified that KAT2A and HAT1 showed significantly differential expression, which was consistent with CNV variation in in 6 urological cancer types (**Figures 2E, F**). However, KAT2A was not significantly up-regulated in 800 samples based on GEO and EBI database (**Figure 3A** and **Table S4**), and EP300 was significantly up-regulated in kidney cancer, PRAD and TGCT (**Figure 3B**). To further validate the expression of EP300 in urological cancers, immunohistochemistry analysis showed that EP300 was significantly up-regulated in TGCT (100%, n = 12), kidney_tumor (100%, n = 11), BLCA (99%, n = 12) and PRAD (100%, n = 12) (**Figures 3C, D**). These results indicate that genetic and expression alteration landscape of lysine acetylation regulators across urological cancer types, suggesting that dysregulation of lysine acetylation regulator is involved in urological cancer contexts.

### Oncogenic Pathways Regulated by Lysine Acetylation Regulators

**Figure 4:**
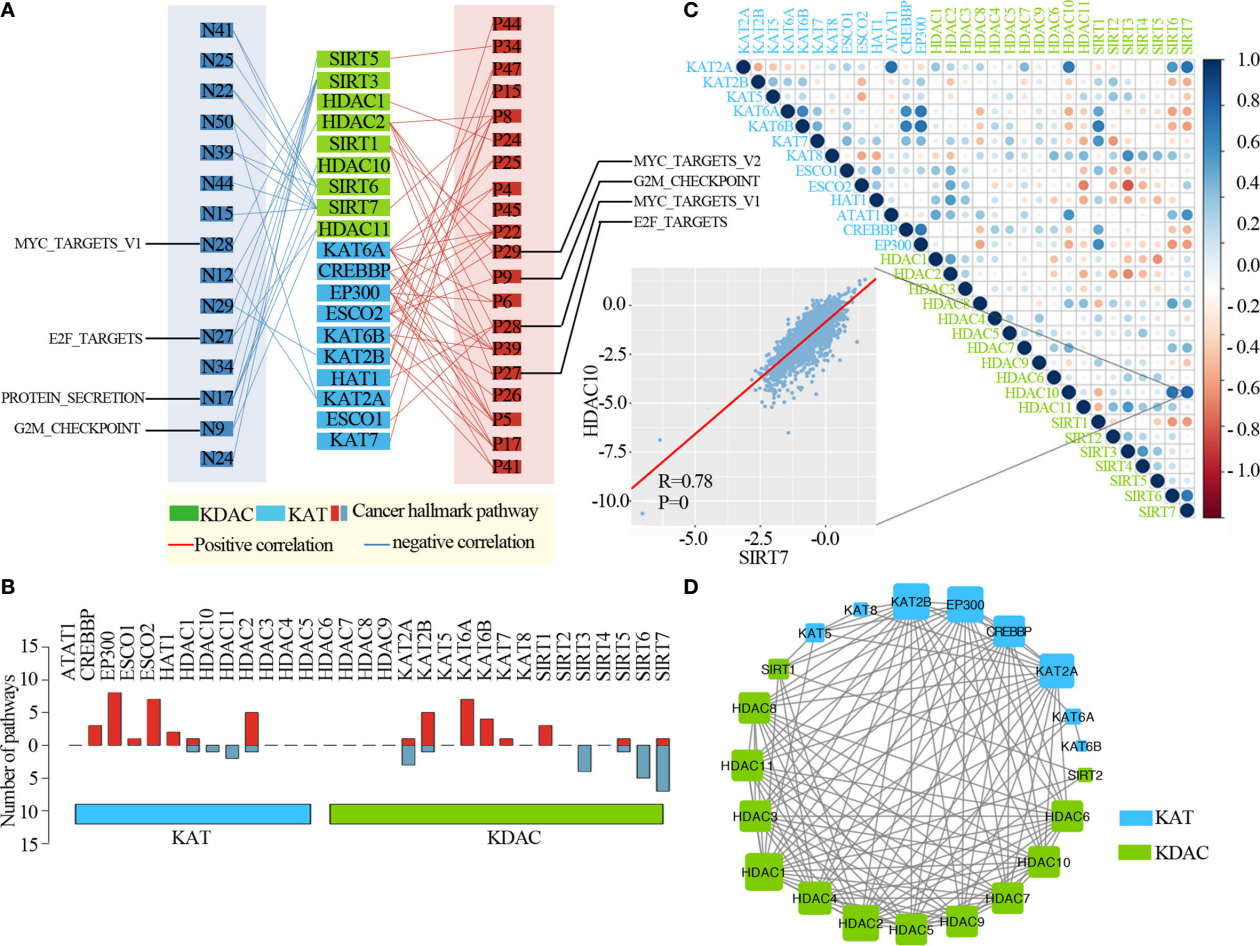
Lysine acetylation regulators are associated with the activation and inhibition of cancer pathways. **(A)** Network diagram demonstrating the correlation between lysine acetylation regulators and cancer pathways. Red represents a positive correlation, and blue represents a negative correlation. The size of the nodes corresponds to the number of links. **(B)** The number of pathways is correlated with individual lysine acetylation regulators. The upper panel is for positively correlated pathways, and the bottom panel is for negatively correlated pathways. **(C)** Correlation among the expression of lysine acetylation regulators. The scatter plot shows the correlation between HDAC10 and SIRT7. **(D)** The protein-protein interactions among lysine acetylation regulators.

To further clarify the molecular mechanisms by which lysine acetylation regulators are involved in cancer, we examined the correlation between the expression of individual lysine acetylation regulators and the activity of 50 cancer hallmark-related pathways. We identified that the expression of lysine acetylation regulators is associated with the inhibition or activation of multiple oncogenic pathways (**Figure 4A** and **Table S3**). The expression of KAT2A, KAT2B, SIRT3, SIRT5, SIRT6 and SIRT7 in KDACs, HDAC1, HDAC2, HDAC10 and HDAC11 in KATs were negatively correlated with a higher number of activated pathways, such as the MYC_targets, E2F_targets, Protein secretion and G2M checkpoint. In particular, we found that the EP300, ESCO2 and HDAC2 in KATs were correlated with the activation of several pathways (**Figure 4B**). Meanwhile, different KATs or KDACs were associated with distinct cancer pathway alterations, suggesting different functional effects of lysine acetylation regulators within the same functional class.

Moreover, to know the interaction of genetic alterations and expression correlation among lysine acetylation regulators, we found not only that genes within the same functional class showed significant co-occurrences of genetic alterations and highly correlated expression patterns, but that a high correlation also existed among KATs and KDACs (**Figure 4C**). For instance, the acetyltransferase KAT6B was significantly correlated with other acetyltransferases, such as, CREBBP and EP300. We also found that there were higher correlations among genes in the same protein complex, such as HDAC10 and SIRT7 (**Figure 2C**, R = 0.78 and P = 0). Meanwhile, we found that these KATs and KDACs interacted with each other frequently in protein-protein interaction networks (**Figure 4D**). There were an especially high number of interactions among the lysine acetylation regulators. Taken together, these results suggest that cross-talk among the KATs and KDACs of lysine acetylation, also mediates the abnormal expression of lysine acetylation regulators and plays critical roles in the development and progression of urological cancers.

### Clinical Relevance of Lysine Acetylation Regulators Across Cancer Types

**Figure 5:**
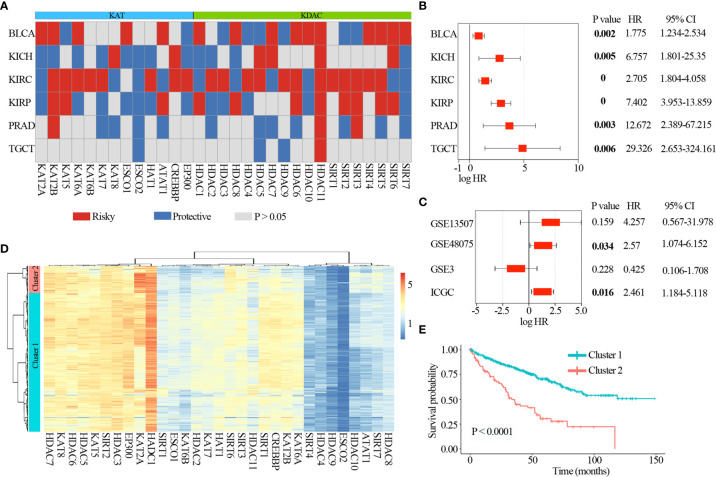
Clinical relevance of lysine acetylation regulators across 6 cancer types. **(A)** Summary of the correlation between expression of lysine acetylation regulators and patient survival. Red represents a higher expression of lysine acetylation regulator associated with worse survival, and blue represents an association with better survival. Only p values < 0.05 are shown. **(B)** The distribution of hazard ratios across 6 cancer types. **(C)** The distribution of hazard ratios across different GEO datasets. **(D)** Heat map showing the clustering for kidney renal clear cell carcinoma patients based on the expression of lysine acetylation regulators. **(E)** Kaplan-Meier survival plot of patients grouped by global expression pattern of lysine acetylation regulators.

To further explore the clinical relevance of lysine acetylation regulators, we first analyzed prognostic value of lysine acetylation regulators in urological cancers. We found that all of the lysine acetylation regulators were associated with the overall survival of patients in at least one cancer type (**Figure 5A**). Several lysine acetylation regulator genes showed oncogenic features, such as HDAC11 and SIRT4, and higher expression of these genes was associated with worse survival across cancer types.

In particular, high expression of HDAC11 was correlated with worse survival in 6 cancer types (**Figure 5B**), including BLCA (log-rank *P* = 0.002), KICH (log-rank *P* = 0.005), KIRC (log-rank *P* = 0), KIRP (log-rank *P* = 0), PRAD (log-rank *P* = 0.003) and TGCT (log-rank *P* = 0.006). Moreover, we collected another 4 datasets across three tissues from Gene Expression Omnibus (GEO) and International Cancer Genome Consortium (ICGC), and found that high expression of HDAC11 was associated with poor patient survival in GSE48075 and RECA-EU (ICGC) (**Figure 5C**). These observations indicate that HDAC11 might function as an oncogene across cancer types. In contrast, we found that several lysine acetylation regulators also showed features of tumor suppressors, such as ESCO2. Higher expression of ESCO2 was significantly associated with better survival in five cancer types.

**Figure 6:**
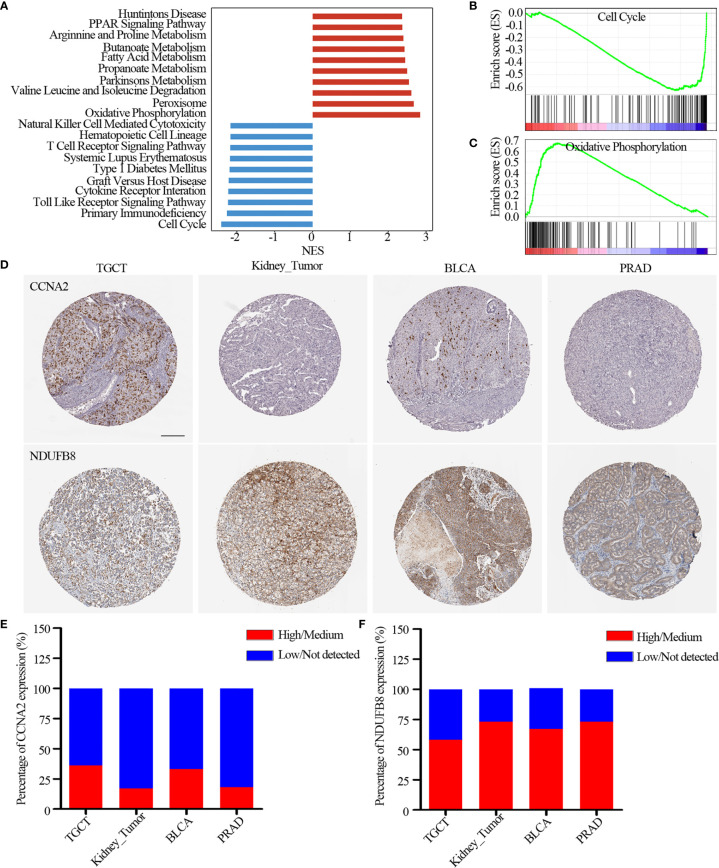
Pathways potentially regulated by HDAC11. **(A)** Distribution of normalized enrichment scores for pathways. The pathways colored in blue was the depleted pathways in HDAC11 downregulation, while the red one is enriched pathway. **(B, C)** GSEA-enrichment plot of the representative gene sets. **(B)** Cell cycle; **(C)** Oxidative phosphorylation. **(D)** Immunohistochemistry images of CCNA2 and NDUFB8 in TGCT, kidney_tumor, BLCA and PRAD. Scar bar = 200um. **(E, F)** Protein expression percentage of CCNA2 **(E)** and NDUFB8 **(F)** analyzed by immunohistochemistry.

Moreover, we found lysine acetylation regulators that were associated with patient survival in KIRC. We thus explored whether the expression of lysine acetylation regulators could contribute to the stratification of kidney cancer. Based on the global expression pattern of lysine acetylation regulators, we identified two subgroups of kidney cancer patient (**Figure 5D**). The first subgroup consisted of 441 patients that showed higher expression of lysine acetylation regulators (Cluster 1), and the second of 86 patients with low expression (Cluster 2). Compared to the Cluster 2 subgroup, patients in the Cluster 1 subgroup had significantly better survival rates (**Figure 5E**, log-rank *P* < 0.0001). To further validate the clinical implications of lysine acetylation regulators, based on the mRNA expression of HDAC11 in KIRC, gene set enrichment analysis (GSEA) was performed to identify pathways potentially linked to HDAC11. Pathway analysis based on the KEGG database was performed, which identified 20 pathways with significant differences in gene expression (P < 0.05) (**Figure 6A**). GSEA analysis showed that hallmarks of cell cycle and oxidative phosphorylation were significantly enriched (**Figures 6B, C**). To further explore the activity of cell cycle and oxidative phosphorylation, the expression of CDK2 and CCNA2 in cell cycle pathway and the expression of NDUFB8 and SDHB in oxidative phosphorylation pathway were analyzed. The results showed that the high and medium expression percentage of CCNA2 in TCGT (36%, n = 11), kidney_tumor (17%, n = 12), BLCA (33%, n = 12) and PRAD (18%, n = 11) (**Figures 6D, E**), CDK2 in TCGT (0%, n = 11), kidney_tumor (0%, n = 12), BLCA (1%, n = 11) and PRAD (0%, n = 10) (**Figures S3A, B**) were up-regulated, while, the high and medium expression percentage of NDUFB8 in TCGT (58%, n = 12), kidney_tumor (73%, n = 11), BLCA (67%, n = 12) and PRAD (73%, n = 11) (**Figures 6D, F**), SDHB in TCGT (100%, n = 10), kidney_tumor (83%, n = 12), BLCA (100%, n = 9) and PRAD (100%, n = 11) (**Figures S3A, C**) were up-regulated. The results indicated the cell cycle was significantly inactivated, however, oxidative phosphorylation was significantly activated in urological cancers. Together, these results suggest a diverse potential of lysine acetylation regulators in the prognostic stratification of specific types of urological cancer and in the development of targeted treatment strategies.

## Discussion

This study demonstrates the prevalent genetic and expression alterations of lysine acetylation regulators across urological cancer types. These lysine acetylation regulators are significantly correlated with the activation and inactivation of cancer pathways, and are also associated with prognostically urological cancers. These results provide new mechanistic understanding of lysine acetylation regulators in urological cancers.

KIRC, BLCA and PRAD are the most common urological cancers (25, 26). Despite improved primary prevention, detection, and treatment, the incidence of age-related cancers of the urinary tract is likely to rise as a result of global population ageing (27, 28). Therefore, it is vital to identify and address the most relevant perturbed genes/proteins for further early detection, investigation, and therapy of urological malignancies.

Dysfunctions in epigenetic and genomics regulation play critical roles in tumor development and progression. KATs and KDACs are functionally opposing epigenetic regulators, which control the activation status of tumor suppressor genes or oncogenes. Upregulation of HDAC activities could result in silencing of tumor suppressor genes and uncontrolled malignant characteristics in urological tumors (29–32). In this study, we comprehensively and systematically explored the genetic alterations and expression perturbations of KATs and KDACs. And we found that the mutation frequency of 31 lysine acetylation regulators, except for CREBBP and EP300, was completely low in KIRP, PRAD, BLCA and KIRC. CNV amplification of acetylation regulators were significantly increased in TCGT, KIRP, KIRC, BLCA and PRAD, while CNV deletion in KICH were found. However, the CNV of HDAC8 and HDAC6 were unchanged in urological cancers. Expression analysis indicated that lysine acetylation regulators were downregulated in KICH, which may be associated with the CNV deletion, while upregulated in other 5 urological cancers, which may be associated with CNV amplification in cancers. However, the expression and prognostic roles of acetylation regulators in urological cancers were not completely consistent. Thus, abnormal expression of acetylation regulators was regulated not only CNV, but also interaction network between acetylation regulators. However, the concrete mechanism still need to be further explored.

Histone deacetylation describes the removal of acetyl groups regulated by KDACs. Widespread genetic alterations (including mutations and CNV) in lysine acetylation regulators were significantly associated with the activation of MYC_targets, E2F_targets, Protein secretion and G2M checkpoint. Histone deacetylase11 (HDAC11), one member of the KDACs family, is associated with condensed chromatin structures that in turn suppress transcription. HDAC11 were significantly up-regulated in 6 urological tumors, GSEA analysis found that dysregulation of HDAC11 was involved in cell cycle and oxidative phosphorylation pathways, which was consistent with hallmarks of acetylation regulators and other studies (33–35). Our immunohistochemistry analysis also validated that cell cycle pathway (CDK2 and CCNA2) was significantly inactivated and oxidative phosphorylation pathway (NDUFB8 and SDHB) was significantly activated, which would may be associated with abnormal expression of acetylation regulators in urological cancers. However, the concrete mechanism of HDAC11 on urological tumors need be further explored.

## Conclusion

In summary, this systematic analysis of the landscape of molecular alterations and clinical relevance of lysine acetylation regulators clarifies a profound understanding the dysregulation of lysine acetylation regulators. It will also provide insights into the development of urological cancers.

## Data Availability Statement

The original contributions presented in the study are included in the article/**Supplementary Material**. Further inquiries can be directed to the corresponding authors.

## Author Contributions

JZ, CZ, HualiJ, HualongJ, and YY conducted and designed experiments, performed data analysis, and drafted the manuscript. HualongJ and YY supervised the project, designed experiments, and edited the manuscript. All authors contributed to the article and approved the submitted version.

## Funding

This study was supported by grants from the National Natural Science Foundation of China (82003212), Discipline construction project of Guangzhou Medical University during the 14th five year plan (06-410-2107181), Guangzhou Key Medical Discipline Construction Project Fund (02-412-B205002-1004042), Guangzhou High Level Clinical Key Specialty Construction Project (2019-2021) and Clinical Key Specialty Construction Project of Guangzhou Medical University (YYPT202017).

## Conflict of Interest

The authors declare that the research was conducted in the absence of any commercial or financial relationships that could be construed as a potential conflict of interest.

## Supplementary Material

Supplementary Figure 1 Mutation frequency distribution of lysine acetylation regulators across different urological cancer types. Left circos plot **(A)** showing the mutation frequency of lysine acetylation regulators in CCLE, and right circos **(B)** showing the mutation frequency in GDSC. Each circos represents one cancer type, which were shown in the bottom panel. *P < 0.05.

Supplementary Figure 2 CNV alterations of lysine acetylation regulators across cell lines in different urological cancer types. Left circos plot **(A)** showing the CNV frequency of lysine acetylation regulators in CCLE, and right circos **(B)** showing the CNV frequency in GDSC. Each circos represents one cancer type, which were shown in the bottom panel.

Supplementary Figure 3
**(A)** Immunohistochemistry images of CDK2 and SDHB in TGCT, kidney_tumor, BLCA and PRAD. Scar bar = 200um. **(B, C)** Protein expression percentage of CDK2 **(B)** and SDHB **(C)** analyzed by immunohistochemistry.
