# Supplementary figures and images for: Molecular Characterization and Clinical Relevance of Lysine Acetylation Regulators in Urological Cancers
Source: Front Oncol. 2021 May 31;11:647221. doi: 10.3389/fonc.2021.647221 (PMC8202406; doi:10.3389/fonc.2021.647221)

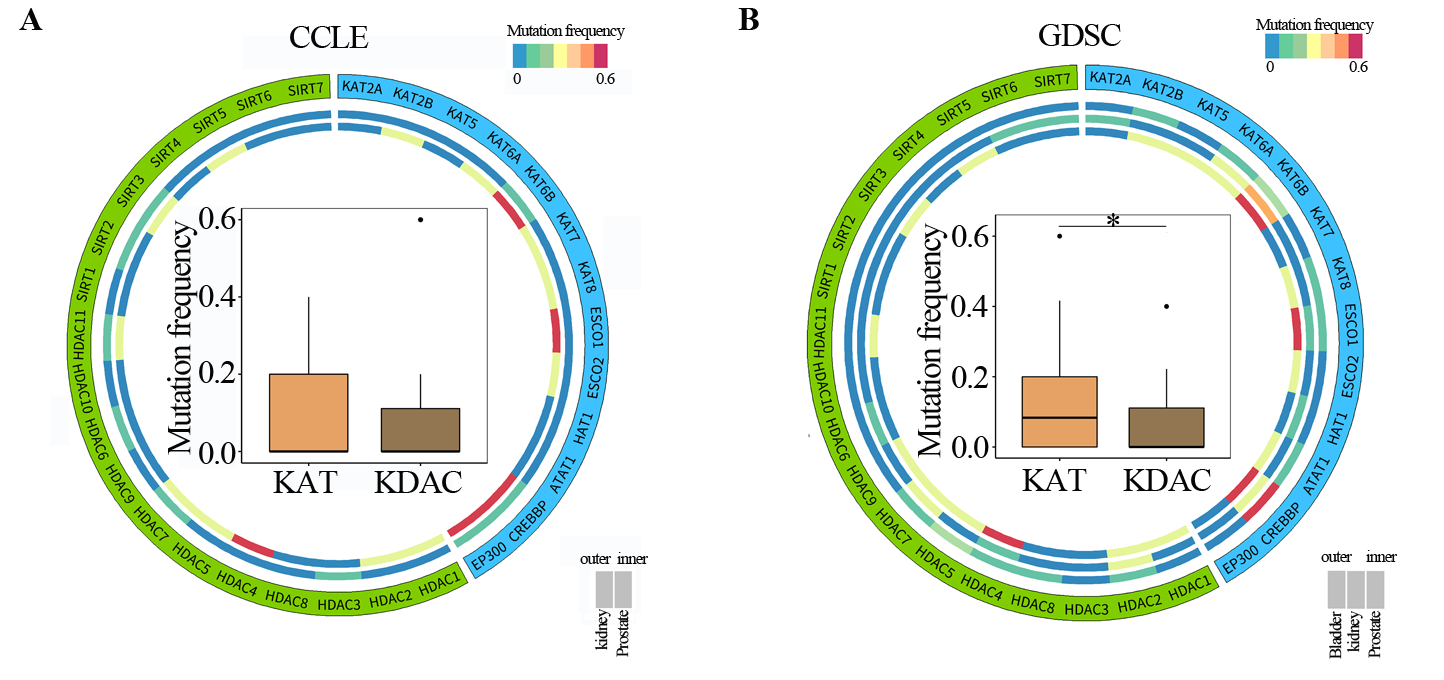

Supplement: Supplementary Figure 1 — Mutation frequency distribution of lysine acetylation regulators across different urological cancer types. Left circos plot (A) showing the mutation frequency of lysine acetylation regulators in CCLE, and right circos (B) showing the mutation frequency in GDSC. Each circos represents one cancer type, which were shown in the bottom panel. *P < 0.05. [file Image_1.tif]

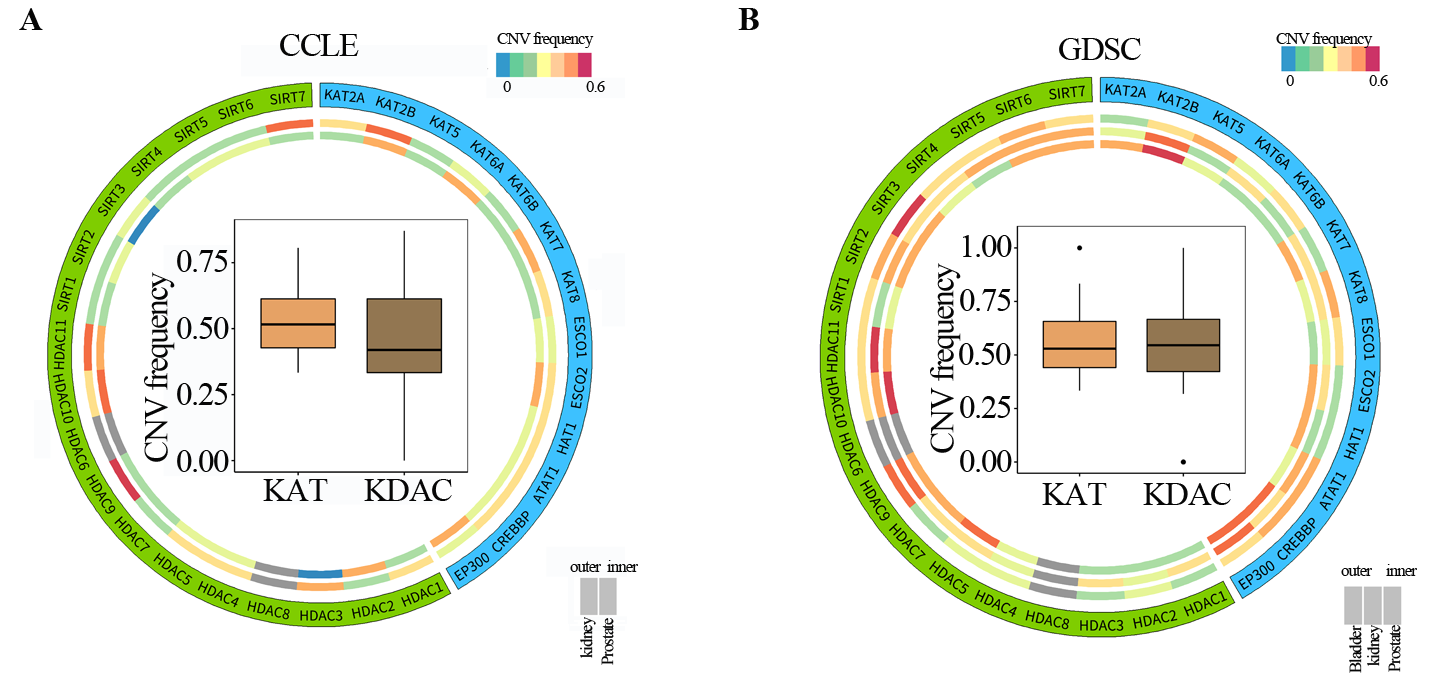

Supplement: Supplementary Figure 2 — CNV alterations of lysine acetylation regulators across cell lines in different urological cancer types. Left circos plot (A) showing the CNV frequency of lysine acetylation regulators in CCLE, and right circos (B) showing the CNV frequency in GDSC. Each circos represents one cancer type, which were shown in the bottom panel. [file Image_2.tif]

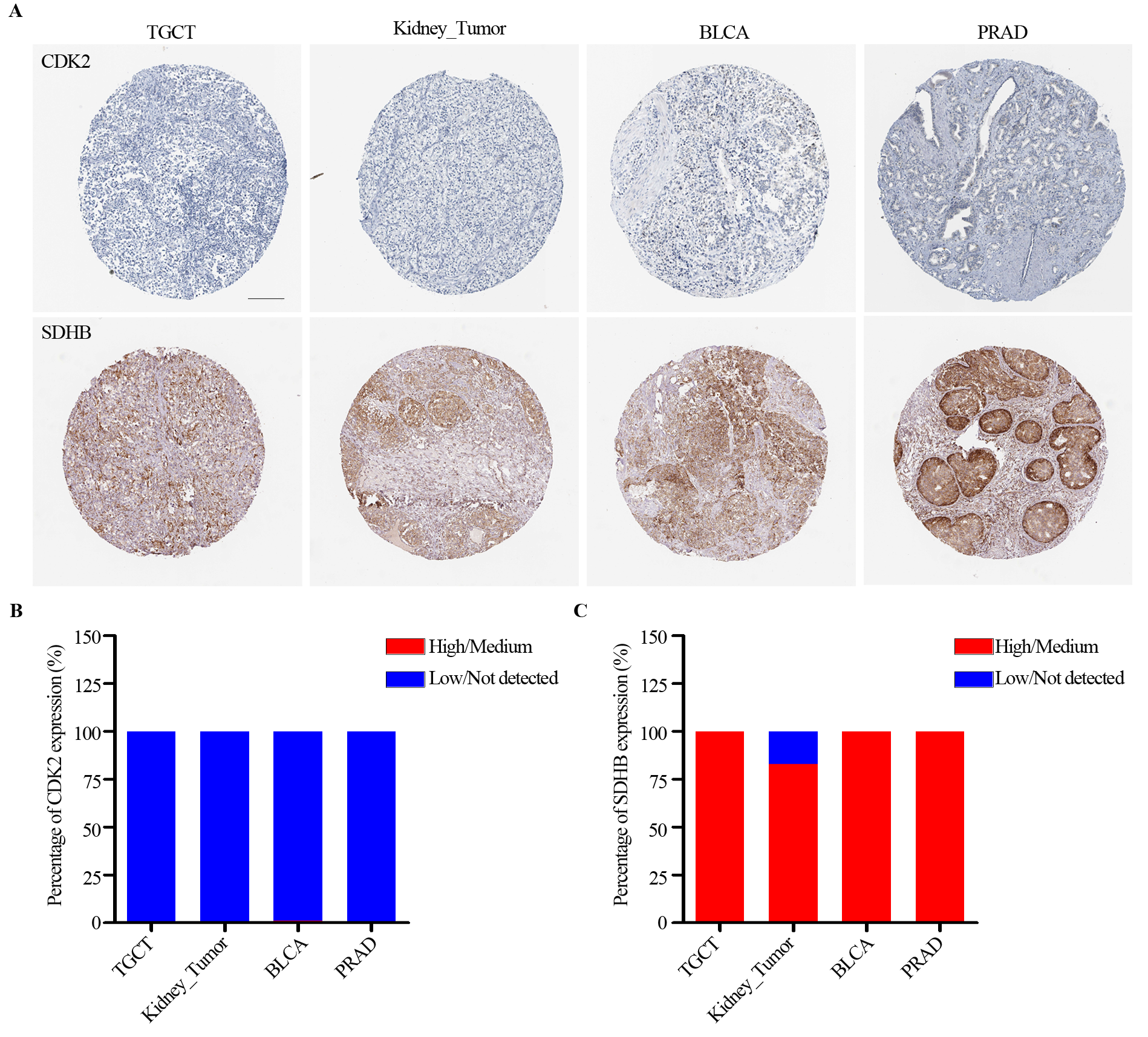

Supplement: Supplementary Figure 3 — (A) Immunohistochemistry images of CDK2 and SDHB in TGCT, kidney_tumor, BLCA and PRAD. Scar bar = 200um. (B, C) Protein expression percentage of CDK2 (B) and SDHB (C) analyzed by immunohistochemistry. [file Image_3.tif]
